# Quantification of bulk lipid species in human platelets and their thrombin-induced release

**DOI:** 10.1038/s41598-023-33076-4

**Published:** 2023-04-15

**Authors:** Susanne Heimerl, Marcus Höring, Dominik Kopczynski, Alexander Sigruener, Christina Hart, Ralph Burkhardt, Anne Black, Robert Ahrends, Gerhard Liebisch

**Affiliations:** 1 Institute of Clinical Chemistry and Laboratory Medicine, University Hospital Regensburg, Franz-Josef-Strauss-Allee 11, 93042 Regensburg, Germany; 2 Department of Analytical Chemistry, University of Vienna, Vienna, Austria; 3 Department of Hematology and Oncology, Internal Medicine III, University Hospital Regensburg, Regensburg, Germany

**Keywords:** Membrane lipids, Diagnostic markers, Lipidomics, Glycerides

## Abstract

Lipids play a central role in platelet physiology. Changes in the lipidome have already been described for basal and activated platelets. However, quantitative lipidomic data of platelet activation, including the released complex lipids, are unavailable. Here we describe an easy-to-use protocol based on flow-injection mass spectrometry for the quantitative analysis of bulk lipid species in basal and activated human platelets and their lipid release after thrombin activation. We provide lipid species concentrations of 12 healthy human donors, including cholesteryl ester (CE), ceramide (Cer), free cholesterol (FC), hexosylceramide (HexCer), lysophosphatidylcholine (LPC), lysophosphatidylethanolamine (LPE), phosphatidylcholine (PC), phosphatidylethanolamine (PE), phosphatidylinositol (PI), phosphatidylserine (PS), sphingomyelin (SM) and triglycerides (TG). The assay exhibited good technical repeatability (CVs < 5% for major lipid species in platelets). Except for CE and TG, the inter-donor variability of the majority of lipid species concentrations in platelets was < 30% CV. Balancing of concentrations revealed the generation of LPC and loss of TG. Changes in lipid species concentrations indicate phospholipase-mediated release of arachidonic acid mainly from PC, PI, and PE but not from PS. Thrombin induced lipid release was mainly composed of FC, PS, PC, LPC, CE, and TG. The similarity of the released lipidome with that of plasma implicates that lipid release may originate from the open-canalicular system (OCS). The repository of lipid species concentrations determined with this standardized platelet release assay contribute to elucidating the physiological role of platelet lipids and provide a basis for investigating the platelet lipidome in patients with hemorrhagic or thrombotic disorders.

## Introduction

Platelets play a central role in hemostasis and thrombosis. When coagulation is initiated by endothelial damage, platelets adhere to the injury site via the von Willebrand factor. Subsequent platelet activation includes platelet shape change and release of hemostatic agonists from platelet granules (reviewed in^1^). Moreover, upon platelet activation, phosphatidylethanolamine (PE) and phosphatidylserine (PS) are translocated to the outer leaflet of the plasma membrane, facilitating coagulation factor interaction on negatively charged surfaces^2,3^. Not only because of this essential physiological process, platelet lipids have been an increasing subject of research for many years. In the 1980s, in particular, phospholipid metabolism was studied in basal and activated platelets. For phosphatidylinositol (PI), a rapid decrease after thrombin-stimulation and subsequent re-accumulation was observed^4–7^. In addition to PI, plasma membrane phosphatidylcholine (PC) and PE were shown to decrease in thrombin-stimulated platelets while the amounts of PS and sphingomyelin (SM) remained unaffected^4^. Later studies utilizing the analytical power of electrospray ionization mass spectrometry focused on the molecular phospholipid species in this context. While PI and PS molecular species profiles were not altered during thrombin and collagen-induced hydrolysis, hydrolysis of arachidonic acid (AA)-containing species was shown for PC and PE^8^. PE plasmalogens were identified as the largest source of AA-release during platelet thrombin stimulation^9^. AA is used to generate various oxylipins, which are essential lipid mediators investigated in detail in multiple studies^10–13^.

Recently, Peng et al.^11^ developed a comprehensive quantitative lipid analysis of murine basal and activated platelets, providing concentrations of almost 400 lipid species and confirming the key findings from human platelets. Their data showed that less than 20% of the lipidome is altered by activation, mainly involving AA-containing lipids. Similarly, Cebo et al. investigated the human platelet lipidome before and upon thrombin-activation^14^. These studies provide a comprehensive set of quantitative data constituting a basis for further understanding the role of platelet lipids. However, quantitative data, including lipid release, are not available.

The current study aimed to quantify the bulk lipidome of human platelets and its thrombin-induced changes, including the release of lipids. Therefore, we established an easy-to-use protocol based on flow-injection analysis mass spectrometry (FIA-MS) for the reproducible and quantitative analysis of bulk lipid species in basal and activated human platelets and their lipid release. The assay was subsequently applied to analyze the concentrations of lipid species in 12 healthy donors.

## Materials and methods

### Blood sampling

Human platelets were obtained from healthy volunteers. Donors did not use non-steroidal anti-inflammatory drugs for at least 10 days. Venous whole blood was collected into 3.2% sodium citrate tubes (Sarstedt, Nümbercht, Germany). The study was approved by the local Ethics Committee (Universitätsklinikum Regensburg, Ethikkommission der medizinischen Fakultät, proposal 17-696-101). All subjects had given their informed consent to participate in the study. All research was performed in accordance with the relevant guidelines and regulations.

### Platelet isolation

Platelet-rich plasma (PRP) was obtained by centrifugation (139 g for 10 min). Platelets were obtained by centrifugation at 900 g for 10 min and at 800 g for 10 min and resuspension in calcium‐free Tyrode‐Hepes buffer (138 mmol/L NaCl, 3 mmol/L KCl, 12 mmol/L NaHCO_3_, 0.4 mmol/L NaH_2_PO_4_, 1 mmol/L MgCl_2_, 5 mmol/L glucose, 10 mmol/L Hepes, 10 mmol/L ethylenediaminetetraacetic acid, 0.5% [wt/vol] bovine serum albumin [BSA]), pH 7.4, sterile‐filtered). The cell number of separated platelets was quantified by a hematology analyzer (XN-350, Sysmex Corporation, Kobe, Japan) and was adjusted to 100/nL. 1 mL of the adjusted platelets was frozen immediately at -80 °C and 1 mL was stimulated by thrombin described below.

### Platelet activation

One milliliter (corresponding to 100 × 10^6^ platelets) of the adjusted platelets was activated by incubation with 1 U/mL bovine thrombin for 15 min at 37 °C. After stimulation, platelets were pelleted by centrifugation at 5000 g for 5 min and 10,000 g for 1 min and resuspended in Tyrode‐Hepes buffer. The supernatant and resuspended pellet were stored at − 80° until lipid analysis.

### Lipid extraction

Lipid extraction was performed according to the method of Bligh and Dyer^15^ in the presence of not naturally occurring lipid species as internal standards. The following lipid species were added as internal standards: PC 14:0/14:0, PC 22:0/22:0, PE 14:0/14:0, PE 20:0/20:0 (di-phytanoyl), PS 14:0/14:0, PS 20:0/20:0 (di-phytanoyl), PI 17:0/17:0, LPC 13:0, LPC 19:0, LPE 13:0, SM 18:1;O2/12:0, Cer 18:1;O2/14:0, Cer 18:1;O2/17:0, GlcCer 18:1;O2/12:0, GlcCer 18:1;O2/17:0, FC[D7], CE 17:0, CE 22:0, TG 51:0, TG 57:0, DG 28:0 and DG 40:0. Platelet homogenates and lipid corresponding to 40 × 10^6^ platelets were extracted. Chloroform phase was recovered by a pipetting robot (Genesis RSP 150, Tecan, Männedorf, Switzerland) and vacuum dried. The residues were dissolved either in 10 mM ammonium acetate in methanol/chloroform (3:1, v/v) (for low mass resolution tandem mass spectrometry) or chloroform/methanol/2-propanol (1:2:4 v/v/v) with 7.5 mM ammonium formate (for high resolution mass spectrometry).

### Lipid mass spectrometry

The analysis of lipids was performed by direct flow injection analysis (FIA) using a triple quadrupole mass spectrometer (FIA-MS/MS; QQQ triple quadrupole) and a hybrid quadrupole-Orbitrap mass spectrometer (FIA-FTMS; high mass resolution).

FIA-MS/MS (QQQ) was performed in positive ion mode using the analytical setup and strategy described previously^16^. A fragment ion of *m/z* 184 was used for lysophosphatidylcholine (LPC)^17^. The following neutral losses were applied: Phosphatidylethanolamine (PE) 141, phosphatidylserine (PS) 185, and phosphatidylinositol (PI) 277^18^. PE-based plasmalogens (PE P) were analyzed according to the principles described by Zemski-Berry^19^. Sphingosine-based ceramides (Cer) and hexosylceramides (HexCer) were analyzed using a fragment ion of *m/z* 264^20^. Quantification was achieved by calibration lines generated by the addition of naturally occurring lipid species to the respective sample matrix. Calibration lines were generated for the following naturally occurring species: LPC 16:0, 18:1, 18:0; PE 34:1, 36:2, 38:4, 40:6 and PE P-16:0/20:4; PS 34:1, 36:2, 38:4, 40:6; Cer 18:1;O2/16:0, 18:0, 20:0, 24:1, 24:0; FC, CE 16:0,18:2,18:1,18:0.

The Fourier Transform mass spectrometry (FIA-FTMS) setup is described in detail in Höring et al.^21^. Triglycerides (TG) and cholesteryl ester (CE) were recorded in positive ion mode FTMS in range *m/z* 500–1000 for 1 min with a maximum injection time (IT) of 200 ms, an automated gain control (AGC) of 1*10^6^, three micro scans and a target resolution of 140,000 (at *m/z* 200). Phosphatidylcholine (PC) and sphingomyelin (SM) were measured in the range *m/z* 520–960. Multiplexed acquisition (MSX) was used for the [M + NH_4_]^+^ of free cholesterol (FC) (*m/z* 404.39) and D7-cholesterol (*m/z* 411.43) with 0.5 min acquisition time, a normalized collision energy of 10%, and IT of 100 ms, AGC of 1*10^5^, isolation window of 1 Da, and a target resolution of 140,000^22^. Data processing details were described in Höring et al.^21^, using the ALEX software^23^ which includes peak assignment and intensity picking.

The extracted data were exported to Microsoft Excel 2016 and processed by self-programmed macros. FIA-FTMS quantification was performed by multiplying the spiked IS amount with the analyte-to-IS ratio. Lipid species were annotated according to the latest proposal for shorthand notation of lipid structures derived from mass spectrometry^24^. For QQQ, glycerophospholipid species annotation was based on the assumption of even-numbered carbon chains only. SM species annotation is based on the assumption that a sphingoid base with two hydroxyl groups is present.

### Statistical analysis

All data are expressed as mean ± SD. Statistical analysis was performed using IBM SPSS statistics 22 software. The t-test determined statistical significance for paired samples. P-values were corrected for multiple testing by the Benjamini–Hochberg procedure. A p-value < 0.05 was considered statistically significant.

### Analysis of lipidome similarities

For the computational comparison of the samples (here termed lipidomes), the lipid names were normalized using the standardization tool Goslin 2.0^25^. In a second step, we put all lipids uniquely in one global list. We pairwisely computed the maximum common subgraph of the lipid chemical structures to achieve a numerical positive distance value between each pair of lipids. Having a list with n lipids, we obtained an n times n matrix. In a consecutive step, we applied a principal component analysis (PCA) to reduce the dimension of this matrix. In the PCA matrix, we split again into several submatrices with respect to the lipids present in each lipidome. We selected each submatrix's first 7 principal components since they already described > 95% of the variance. Additionally, we added the abundances of the corresponding lipids normalized to the variance of the first principal component into each submatrix. Again, we pairwisely compared the distance of all submatrices utilizing the Hausdorff distance. With m lipidomes, we obtained an m times m distance matrix. In the last step, we applied an agglomerative hierarchical clustering with an unweighted average linkage strategy. The resulting clustering is plotted as a dendrogram in Fig. 4.

## Results

### Standardization of platelet release assay

**Figure 1:**
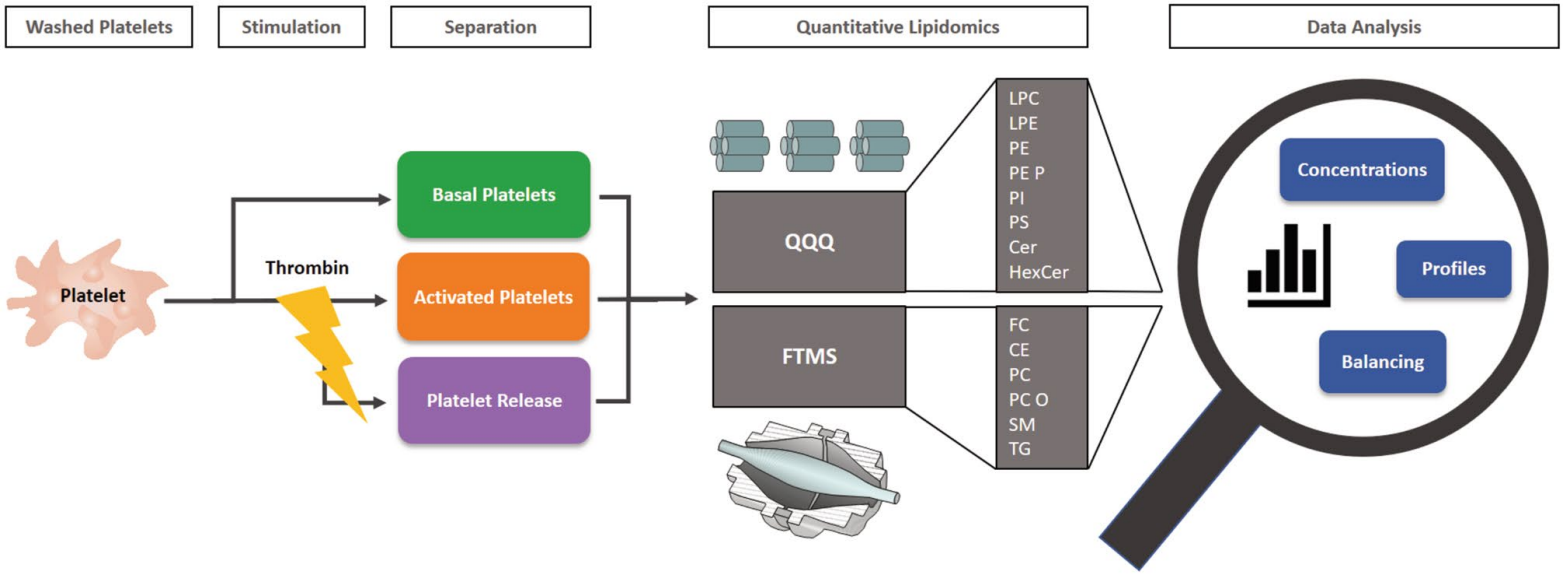
Workflow platelet release assay. Washed platelets were adjusted to a defined cell count (100/nL). One aliquot was stored immediately as basal platelets, a second aliquot was stimulated by thrombin. After a 15 min stimulation and centrifugation, pellet and supernatant were stored as activated platelets and their release, respectively. Samples were analyzed by quantitative lipidomics using flow injection analysis (FIA) coupled with high-resolution mass spectrometry (HRMS) as well as tandem mass spectrometry (MS/MS). The study covered major lipid classes: cholesteryl ester (CE), ceramide (Cer), free cholesterol (FC), hexosylceramide (HexCer), lysophosphatidylcholine (LPC), lysophosphatidylethanolamine (LPE), phosphatidylcholine (PC), phosphatidylcholine ether (PC O), phosphatidylethanolamine (PE), PE plasmalogens (PE P), phosphatidylinositol (PI), phosphatidylserine (PS), sphingomyelin (SM), trigylcerides (TG).

In a first step, we developed a standardized assay to quantify lipid species in platelets before and after thrombin-induced aggregation (Fig. 1). Platelets were separated from whole blood by centrifugation and washing. Washed platelets were adjusted to a defined cell count (100/nL). While one aliquot was stored immediately as basal platelets, a second aliquot was stimulated by thrombin. Since we did not aim to evaluate short-term thrombin action, but were interested in full platelet activation, a stimulation time of 15 min was chosen. After stimulation and centrifugation, pellet and supernatant were stored as activated platelets and their release, respectively. These three samples were subjected to quantitative lipidomics by flow injection analysis (FIA) coupled with high-resolution mass spectrometry (HRMS) and tandem mass spectrometry (MS/MS). The study covered the major lipid classes CE, Cer, FC, HexCer, LPC, LPE, PC, PC O, PE, PE P, PI, PS, SM, TG, and related lipid species. Concentrations were calculated as nmol/10^9^ platelets. Technical reproducibility of the data was demonstrated by triplicate analysis showing a minor CV of < 5% for most lipid classes and the main lipid species in basal and activated thrombocytes (Suppl. Table 1). Variation was higher for neutral lipid classes CE and TG and species and lipid classes close to the limit of detection (LOD).

### Lipid class concentrations of basal and activated platelets

This study aimed to provide a concentration repository for the bulk lipidome of healthy human platelets and their release. Therefore, platelets were purified from 6 healthy male (mean age 42 years) and 6 female (mean age 38.3 years) volunteers who did not receive any non-steroidal anti-inflammatory drugs during the past 10 days. Isolated platelets were subjected to thrombin-induced aggregation and analyzed as described above. We did not observe any relevant gender-specific difference in the platelet lipidome (data not shown). Therefore, we merged the data obtained from male and female donors for all results presented here.

**Figure 2:**
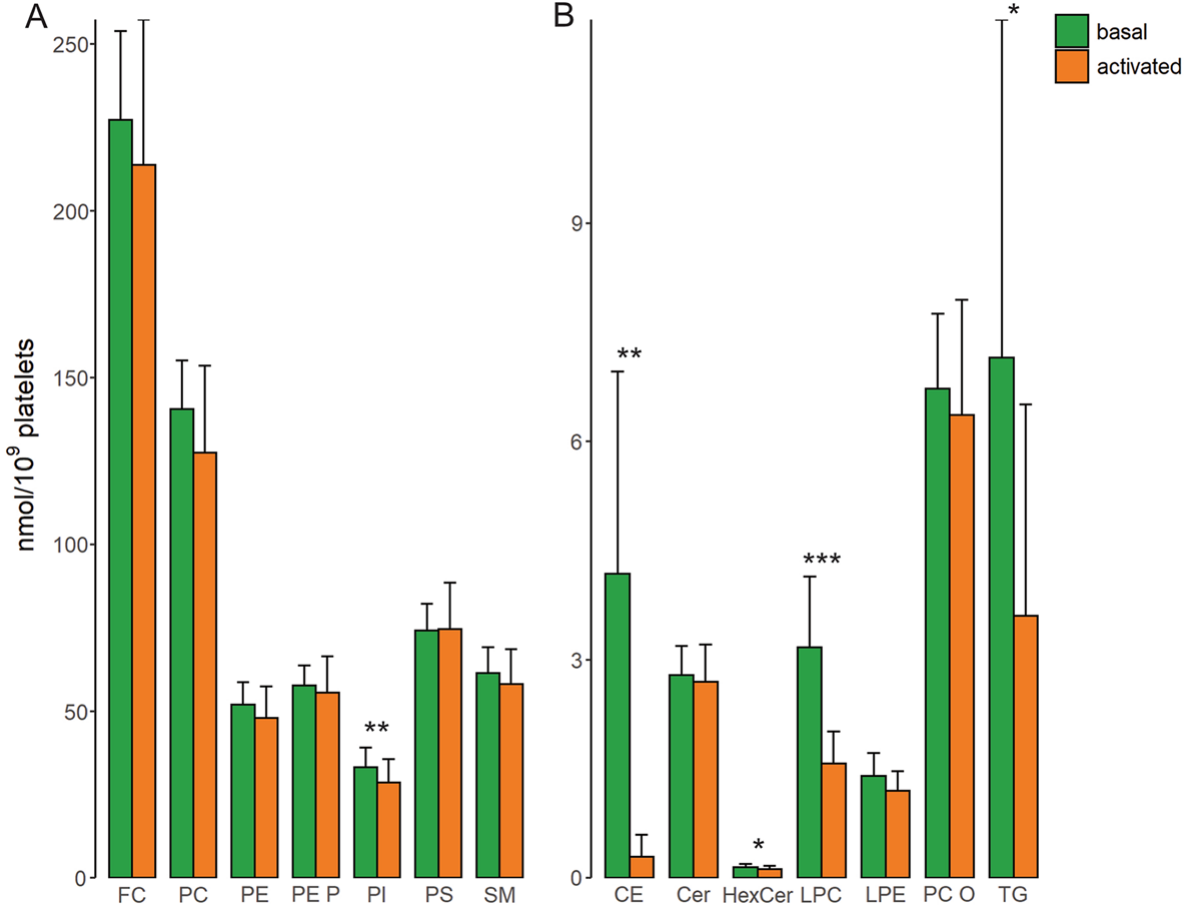
Lipid class concentrations of platelets. Displayed are sum concentrations of lipid classes of unstimulated (basal) and activated platelets upon thrombin stimulation for major (**A**) and minor (**B**) lipid classes. Mean and SD of 12 healthy human donors. *p < 0.05, **p < 0.01, ***p < 0.001.

In a first step, we analyzed the lipid composition of platelets before activation. FC (227 ± 21 nmol/10^9^ platelets), PC (141 ± 15 nmol/10^9^ platelets), and PS (74 ± 8 nmol/10^9^ platelets) were the most abundant lipid classes in basal platelets (green bars in Fig. 2; Suppl. Table 2). Inter-donor variation in basal platelets was below 20% CV for the main lipid classes and up to 30% CV for minor classes. The highest variations with more than 60% CV between donors were observed for CE and TG. Except for these neutral lipids, the variation observed for the main lipid species was also below 30% CV (Suppl. Table 3).

The lipid concentration (Fig. 2) and relative lipid composition of activated compared to basal platelets (Suppl. Figure S1A) did not change substantially for most lipid classes with the exception of CE and TG. For CE and TG we observed a substantial reduction in activated platelets by more than 90% and ~ 50%, respectively. Interestingly, also PI (87% of basal), and the lysophospholipids LPC (50% of basal) and LPE (15% of basal) decreased significantly.

### Quantification of platelet lipid release

**Figure 3:**
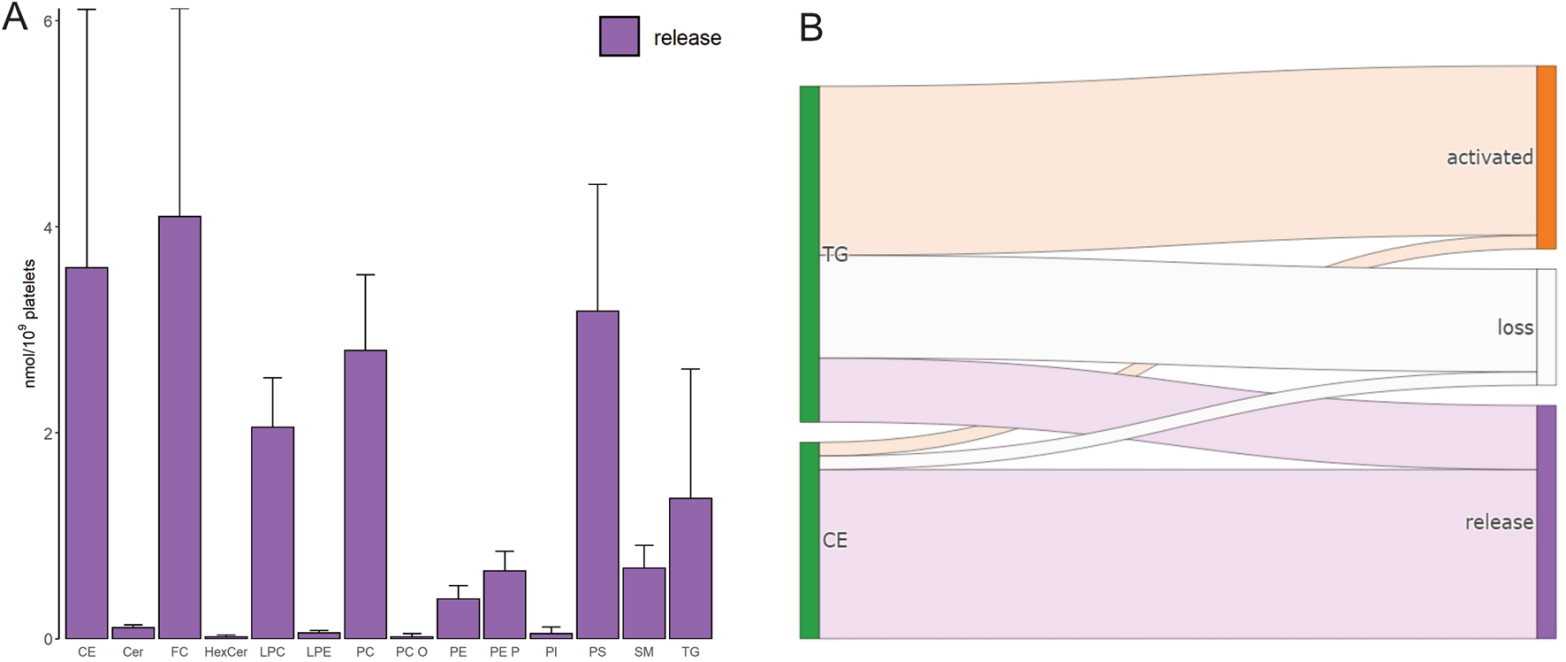
Lipid species release. Displayed are (**A**) sum concentrations of lipid classes of lipid release upon thrombin stimulation. Mean and SD of 12 healthy human donors. (**B**) Sankey diagram showing the lipid flow of neutral lipids triglycerides (TG) and cholesteryl ester (CE) upon platelet activation (mean concentration n = 12).

Beside lipid concentrations of platelets we also quantified the release of lipids in the supernatant of activated platelets. As expected from the minor differences between basal and activated platelets the overall release of lipids was comparatively low: FC (4.1 ± 2.0 nmol/10^9^ platelets), CE (3.6 ± 2.5 nmol/10^9^ platelets), PS (3.2 ± 1.2 nmol/10^9^ platelets), PC (2.8 ± 0.7 nmol/10^9^ platelets), LPC (2.1 ± 0.5 nmol/10^9^ platelets) and TG (1.4 ± 1.2 nmol/10^9^ platelets) (Fig. 3A). Thus, lipid release was composed mainly of FC (21 ± 10%), PS (17 ± 6%) PC (15 ± 5%), LPC (11 ± 2%) and neutral lipids CE (18 ± 11%) and TG (6 ± 6%) (Suppl. Figure S1B).

Together with the platelet lipid concentration, lipid release data permitted an analysis of lipid flow upon platelet activation (Suppl. Table 2). Except for neutral lipids, almost the entire lipid content of basal platelets remained in activated platelets (Fig. 3B). The majority (86%) of CE was found in the lipid release, while half of the basal TG concentration was found in activated platelets and about 20% in the lipid release. Consequently, ~ 30% of the basal TG was presumably subject to metabolism.

### Changes in lipid species concentrations and profiles upon activation of human platelets

**Figure 4:**
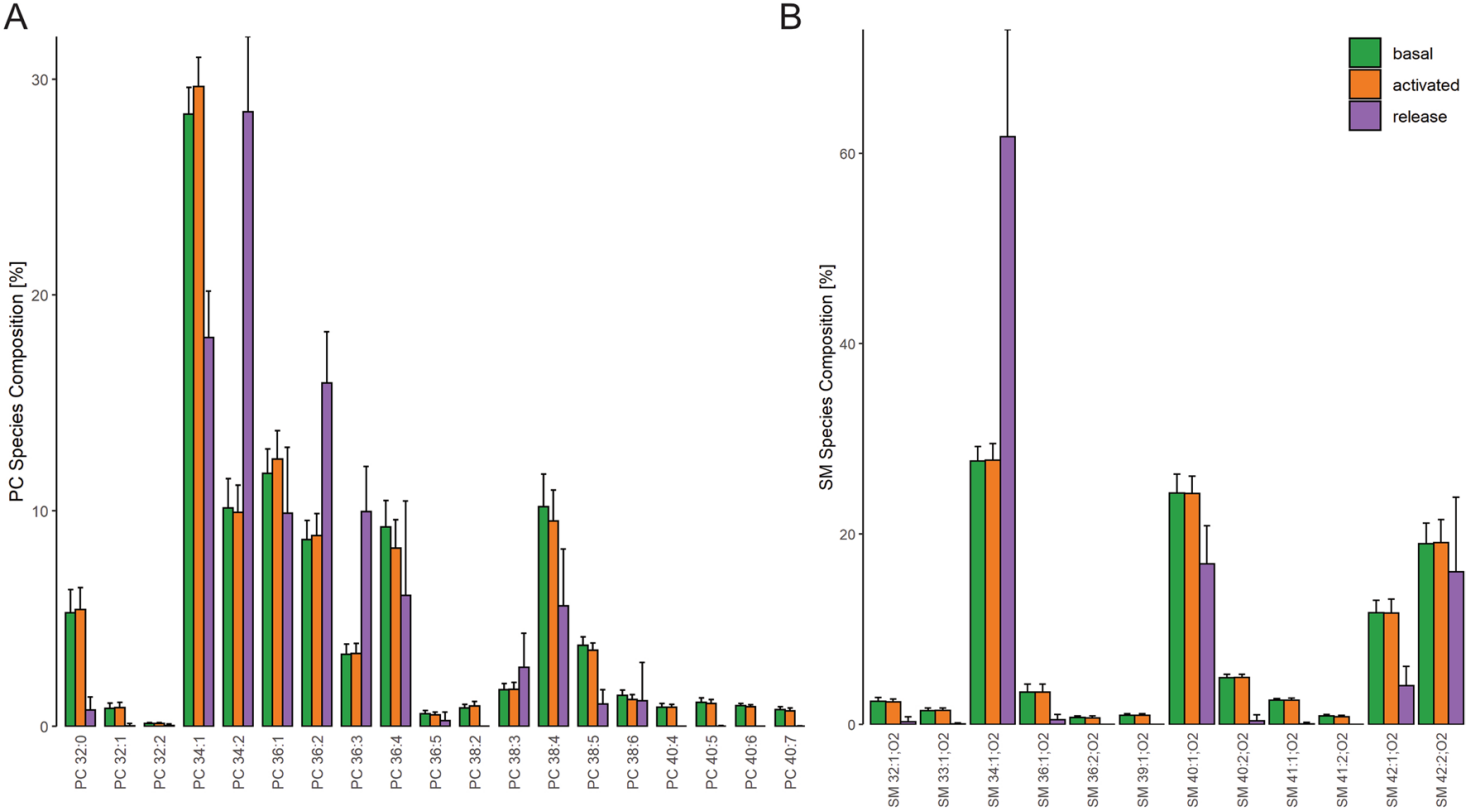
Lipid species profiles of PC and SM. Displayed are (**A**) PC and (**B**) SM lipid species profiles related to the sum of the respective lipid class of unstimulated (basal), activated platelets and lipid release upon thrombin stimulation. Mean and SD of 12 healthy human donors.

Further, we analyzed lipid species concentrations and profiles to check whether specific lipid pools were released or metabolized (Suppl. Figures S2–14). Evaluation of lipid species release is only appropriate when concentrations for most of the released lipid molecules are above LOD and exhibit sufficient precision. Therefore, species profiles for the lipid release were calculated (% of respective lipid class) for PC, SM, LPC, TG, and CE. For PC and SM, the species profiles of lipid release revealed an altered pattern (Fig. 4). While PC 34:1 represented a fraction of about 30% of basal and activated platelets, PC 34:2 was the main species of the released PC species. The main SM species in platelets were SM 34:1;O2, SM 40:1;O2, SM 42:2;O2 and SM 42:1;O2 whereas 60% of the released SM represented SM 34:1;O2.

**Figure 5:**
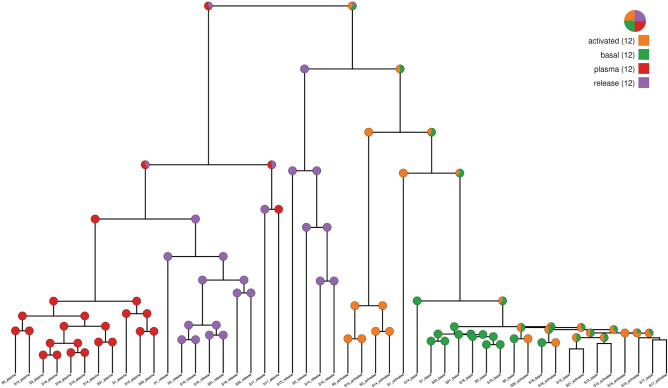
Comparisons of platelet lipidomes with their release and plasma. We pairwisely compared the lipid chemical structures to achieve a numerical positive distance value between each pair of lipids. Having a list with n lipids, we obtained an n times n matrix which was visualized by a principal component analysis considering the lipid quantities. Number of biological replicates n = 12.

To evaluate the similarity of the basal, activated, and released platelet lipidome, we compared all identified and quantified lipids at their individual structural level against each other and to the plasma lipid composition. Surprisingly, if structural distances are considered for clustering, the release and plasma lipidome displayed the highest similarity, indicating a close relationship (Fig. 5).

### Concentration balance indicates phospholipase action

**Figure 6:**
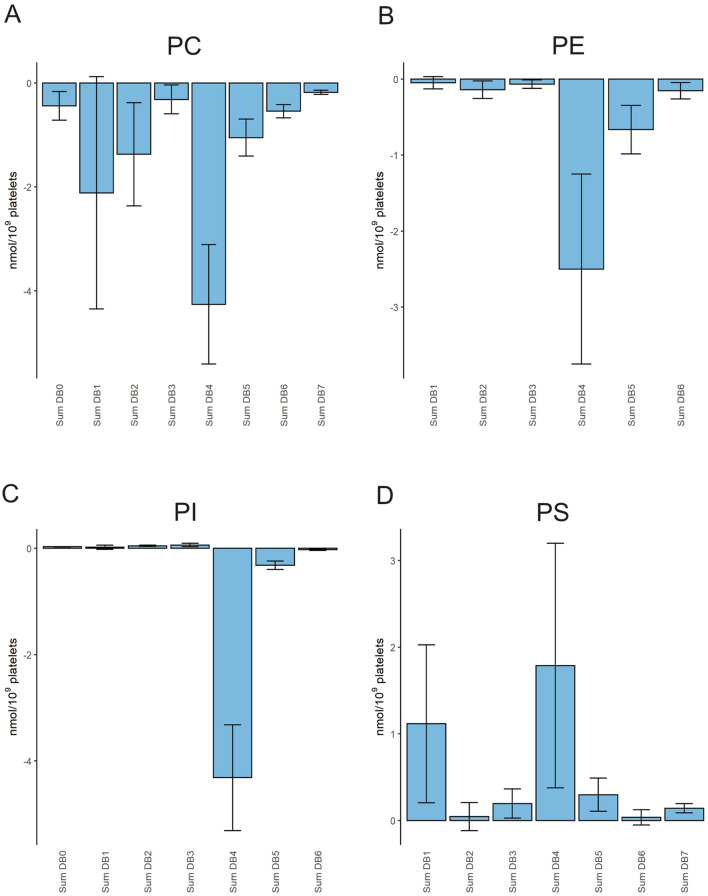
Glycerophospholipid concentration balance after thrombin activation. Concentration balances were calculated as difference of the sum (lipid release and activated platelets) and basal platelets for the following glycerophospholipids: (**A**) PC, (**B**) PE, (**C**) PI and (**D**) PS. Displayed is the sum of species with the respective number of double bonds (DB). Mean and SEM of 12 healthy human donors.

To evaluate the metabolism of lipid species, we subtracted the sum of activated and released lipids from the basal lipid concentrations. Thus, loss or gain in certain lipid species could be easily determined. In particular, we analyzed the release of AA by phospholipase action as a hallmark during platelet activation. Therefore, we compared the concentration balance of different glycerophospholipid classes related to the species’ number of double bonds (DBs) (Fig. 6). Species with four DBs decreased in PC, PE, and PI, while a trend to increased concentrations was found for PS. The loss was most pronounced for PC and PI, with about 4 nmol per billion platelets. It comprised PC 36:4, PC 38:4, and PI 38:4, respectively (Suppl. Figure S15). Like PI, the reduction of ~ 2.5 nmol PE was mainly related to PE 38:4. Beside four DB containing acyl combinations, 16:0/20:4 and 18:0/20:4, AA may be liberated from five DB species representing combinations with 16:1 or 18:1. We observed a loss of ~ 1 nmol for PC and 0.7 nmol for PE. For PC, also species with one and two DBs tended to decrease.

In principle, a phospholipase-mediated loss of glycerophospholipids should be accompanied by an increase in related lysophospholipids. Indeed, saturated LPC showed a small but significant increase of ~ 0.5 nmol/10^9^ platelets (Suppl. Table 2).

### Intra-donor variability of platelet lipidome

Finally, we investigated the range of the biological variability of the platelet lipidome. Therefore, we compared the platelet lipidomes of a male and a female donor at four different time points (within 9 to 15 months). For both donors, concentrations for basal platelets exhibited low variations with CVs ranging from 2 to 30% for most lipid classes (Table 1A). In particular, for neutral lipid classes, high variations were observed with up to 100% for CE and 140% for TG. The results of lipid release are summarized in Table 1B, showing markedly higher CV compared to unstimulated platelets (4–185%).

**Table 1:** Intra-donor variation. Displayed are mean, SD [nmol/10^9^ platelets] and CV (coefficient of variation) for basal platelets (**A**) and lipid release upon thrombin stimulation (**B**).

	Lipid class	CE	Cer	FC	HexCer	LPC	LPE	PC	PC O	PE	PE P	PI	PS	SM	TG
A) Basal platelets															
Male n = 4	Mean	11.72	3.30	207.84	0.21	2.77	1.58	135.94	6.62	46.98	58.78	33.60	67.55	59.25	5.08
	SD	11.79	0.15	45.89	0.00	0.68	0.46	18.50	0.37	3.06	1.86	10.18	4.59	4.97	7.19
	CV [%]	100.52	4.43	22.08	2.23	24.42	29.41	13.61	5.52	6.52	3.16	30.30	6.80	8.39	141.42
Female n = 5	Mean	10.74	3.05	200.37	0.18	2.48	1.26	137.63	6.48	46.54	55.95	29.14	66.98	56.25	6.62
	SD	7.38	0.51	19.42	0.05	0.63	0.35	17.86	0.64	4.24	3.74	7.13	5.90	5.89	3.80
	CV [%]	68.72	16.86	9.69	27.75	25.41	27.73	12.97	9.86	9.12	6.68	24.46	8.81	10.47	57.43
B) Platelet release															
Male n = 4	Mean	10.57	0.13	3.00	0.07	1.25	0.02	7.47	0.03	0.29	0.52	0.13	0.45	1.11	2.77
	SD	10.30	0.02	4.25	0.02	0.05	0.01	6.62	0.01	0.16	0.43	0.07	0.47	0.74	3.92
	CV [%]	97.44	13.64	141.42	27.85	3.90	20.79	88.61	18.80	56.49	83.93	56.03	103.72	66.26	141.42
Female n = 5	Mean	8.34	0.12	3.35	0.04	1.27	0.06	6.71	0.03	0.50	0.71	0.11	1.55	1.08	2.51
	SD	7.88	0.04	3.59	0.04	0.17	0.03	5.52	0.06	0.24	0.13	0.09	0.88	0.70	2.61
	CV [%]	94.56	33.24	107.24	99.53	13.07	61.11	82.34	185.08	48.19	18.54	86.22	57.17	65.09	103.94

## Discussion

A central aim of our study was to establish a quantitative platelet release assay of bulk lipid species based on state-of the art lipidomics and its application in a substantial number of healthy human donors. We demonstrated that the selected stimulation conditions and mass spectrometric analysis by validated FIA-MS methods provides sufficient reproducibility for most lipid species (Suppl Table 1). Besides the robust mass spectrometric analysis, this may also be related to the supra-physiologic thrombin stimulus, similar to various previous studies^7,9,26^, and the stimulation time of 15 min ensuring full activation of platelets and completion of lipidomic alterations. Moreover, analysis of intra-individual CVs showed acceptable biological variation for most lipid classes (Table 1). Highest CVs were observed for the neutral lipids CE and TG, possibly reflecting the variations of open canalicular system (OCS) lipid content (see discussion below). Taken together, the proposed experimental setup, which requires 200 million thrombocytes corresponding to approximately 1 mL citrated whole blood, provides a sound basis for more extensive patient studies and, prospectively, routine testing.

### Basal lipid class concentrations and species profiles

The lipid class composition reported here for platelets showed FC, PC, PE, and PS as the most abundant lipid classes. Similar relative amounts of lipid classes in platelets were described by Leidl et al.^27^ and by Rübsaamen et al. in circulating and in gel filtrated platelets obtained from platelet concentrates^28^, respectively. A similar composition was demonstrated by Peng et al., presenting also quantitative data of murine platelets^11^. In 1996, Han et al. provided mass spectrometry-based quantitative data of phospholipid species of basal and thrombin-activated human platelets^9^. Total PC and PE levels in our study are in very good accordance and even the levels of PE P and PE matched closely. A prior published study applying reverse-phase HPLC to analyze basal and stimulated human platelets reported phospholipid species concentration in a comparable range^8^. Lipid class concentrations determined recently by Cebo et al. in 11 donors^14^ were in a similar range, except more tenfold and sixfold higher CE and SM, respectively.

Lipid species of basal platelets reported here were quite similar to the pattern described by Leidl et al.^27^ as well as by Valkonen et al.^13^ and align reasonably with those reported by Han et al.^9^. Similar species profiles were also reported recently for platelets isolated from healthy subjects and obese patients^29^. However, we could not confirm the presence of short chain PC 18:0, 20:0, 24:0, which were found upregulated in platelets of acute coronary syndrome patients^30^. In summary, good agreement with previous reports supports the validity of the reported concentrations.

### Concentration balance indicate PC, PI, and PE as major source for AA release

Several previous and our studies gave evidence for phospholipase activity releasing AA upon thrombin activation^8,9,11^. The presented release assay permits the balancing of lipid concentrations before and after activation. If species containing 4 and 5 DBs are considered as a source of AA^9^, we found about 5 nmol AA-release per billion platelets from PC and PI, 3 nmol from PE but no release from PS. About a third of the 13 nmol AA per billion platelets was liberated from a single species PI 38:4. Also, Peng et al.^11^ reported PI 38:4 as the major precursor of AA, decreasing 19% upon activation vs. 15% in our study. These data contrast Han et al.^9^, reporting a total AA-release of 60 nmol per billion platelets after 90 s thrombin that originates 53% from PE, 18% from PC, and 15% each from PI and PS. This difference is potentially related to stimulation time. For PI, fast lipolysis within 1 to 2 min was reported to be 60–70% of unstimulated level and re-acylation to ~ 90% after 10 min^5^. Other studies also reported fast phospholipase action on PC, PE, and PI after a few minutes of thrombin stimulation^31,32^. Our study used a longer period of 15 min which could be considered as an endpoint because we aimed to analyze also the overall lipid release. Previous studies investigating short-term thrombin effects usually terminated the reaction by adding organic solvents and did not separate lipid release from activated platelets. Therefore, we cannot rule out that re-acylation of phospholipids occured in our experimental setting, explaining the lower amount of lipolysis in our study as compared to previous studies.

### Released lipid classes and species match lipid profiles in plasma indicating OCS release

Although characterization of the platelet lipidome has been an intensive subject of research since the 1960s (reviewed in^33^), only very limited data on the release of bulk lipids upon platelet activation exist. Notably, the released lipids pattern did not correspond well to the lipid composition of basal platelets. While FC, PC, and PS were found as the main classes in basal platelets, FC, PC, and the neutral lipids, CE and TG were the main lipid classes in the release. These are well-known as major plasma lipids^22,28,34,35^. This might be explained by an evagination of the OCS upon activation^36,37^ accompanied by a release of therein contained plasma lipids into platelet supernatant. Similarity analysis of the lipidomes of basal, activated platelet and release revealed clustering of release and plasma, supporting that the released lipids may originate from OCS.

Valkonen et al. isolated extracellular vesicles from the same platelet concentrate after storage. PC species pattern of these vesicles correlated well with the pattern found in platelet release in our study^13^, showing an increased fraction of PC 34:2, PC 36:2, and PC 36:3. This PC pattern rather match to plasma species profiles of our donors or those reported in previous studies^34,35,38^ than to platelet species profiles. Taken together, these data support that plasma lipids floating into the OCS are ejected upon platelet activation without substantial modification.

### Limitations of the study

This study did not evaluate the short-term effects of thrombin stimulation as most previous studies and, therefore most likely included a re-acylation reaction of glycerophospholipids. However, it is challenging to preserve lipid profiles during centrifugation of stimulated platelets within short stimulation intervals when phospholipase action is high. Another factor, which could be considered in future studies, is whether platelet lipid release is stimulus-dependent e.g. by collagen-related peptide (CRP). Due to the limited number of platelet donors, we could not rule out that there are minor gender differences in the platelet lipidome, for example as reported for plasma lipid lipids^38^.

## Conclusion

The lipid release assay for human isolated platelets, introduced in our study, provides reproducible data. A first repository of 12 healthy donors, including lipid species concentrations of basal and activated platelets and platelet release, provides a profound basis for further elucidation of platelet physiology and pathophysiology like in hemorrhagic and thrombotic disorders.

## Supplementary Information

Supplementary Information 1.

Supplementary Information 2.

Supplementary Information 3.

Supplementary Information 4.

## Data Availability

The data supporting this study are available in the article, the supplementary data or available from the corresponding author upon reasonable request.
