# Supplementary material for: Quantification of bulk lipid species in human platelets and their thrombin-induced release
Source: Sci Rep. 2023 Apr 15;13:6154. doi: 10.1038/s41598-023-33076-4 (PMC10105721; doi:10.1038/s41598-023-33076-4)
Supplement: Supplementary file 1 — Supplementary Information 1. [file 41598_2023_33076_MOESM1_ESM.pdf]

Quantification of bulk lipid species in human platelets and their thrombin-induced release

Susanne Heimerl[1], Marcus Höring[1], Dominik Kopczynski[2], Alexander Sigruener[1], Christina Hart[3], Ralph Burkhardt[1], Anne Black[1], Robert Ahrends[2] and Gerhard Liebisch[1]

[1]Institute of Clinical Chemistry and Laboratory Medicine, University Hospital Regensburg, Germany

[2]Department of Analytical Chemistry, University of Vienna, Austria

[3]Department of Hematology and Oncology, Internal Medicine III, University Hospital Regensburg, Germany

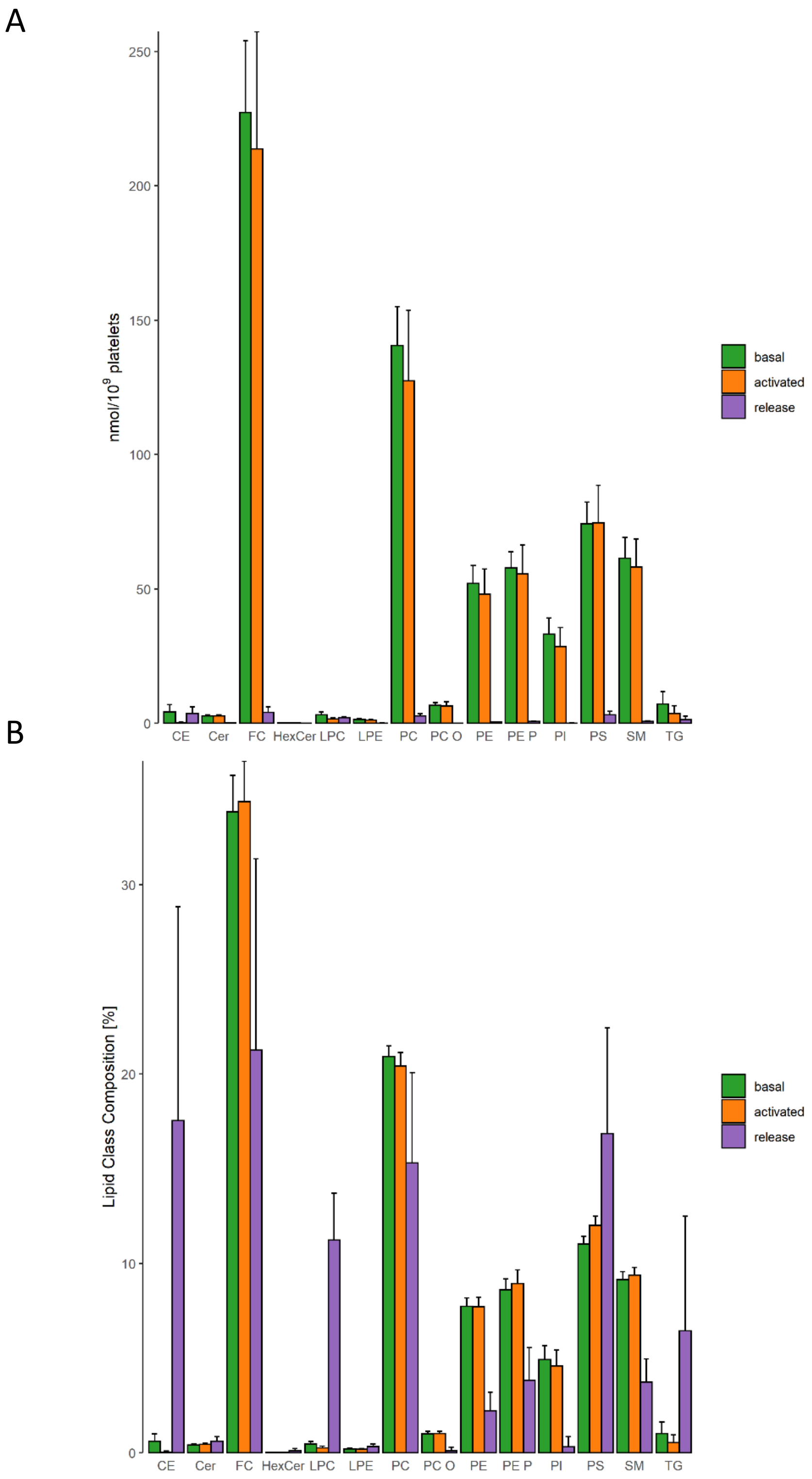

Figure S1. Lipid class concentrations and profiles

Displayed are A) concentrations and B) profiles related to the sum of displayed lipids of unstimulated (basal), activated platelets and lipid release upon thrombin stimulation. Cholesteryl ester (CE), ceramide (Cer), free cholesterol (FC), hexosylceramide (HexCer), lysophosphatidylcholine (LPC), lysophosphatidylethanolamine (LPE), phosphatidylcholine (PC), phosphatidylcholine ether (PC O), phosphatidylethanolamine (PE), PE plasmalogens (PE P), phosphatidylinositol (PI), phosphatidylserine (PS), sphingomyelin (SM), trigylcerides (TG). Mean and SD of 12 healthy human donors.

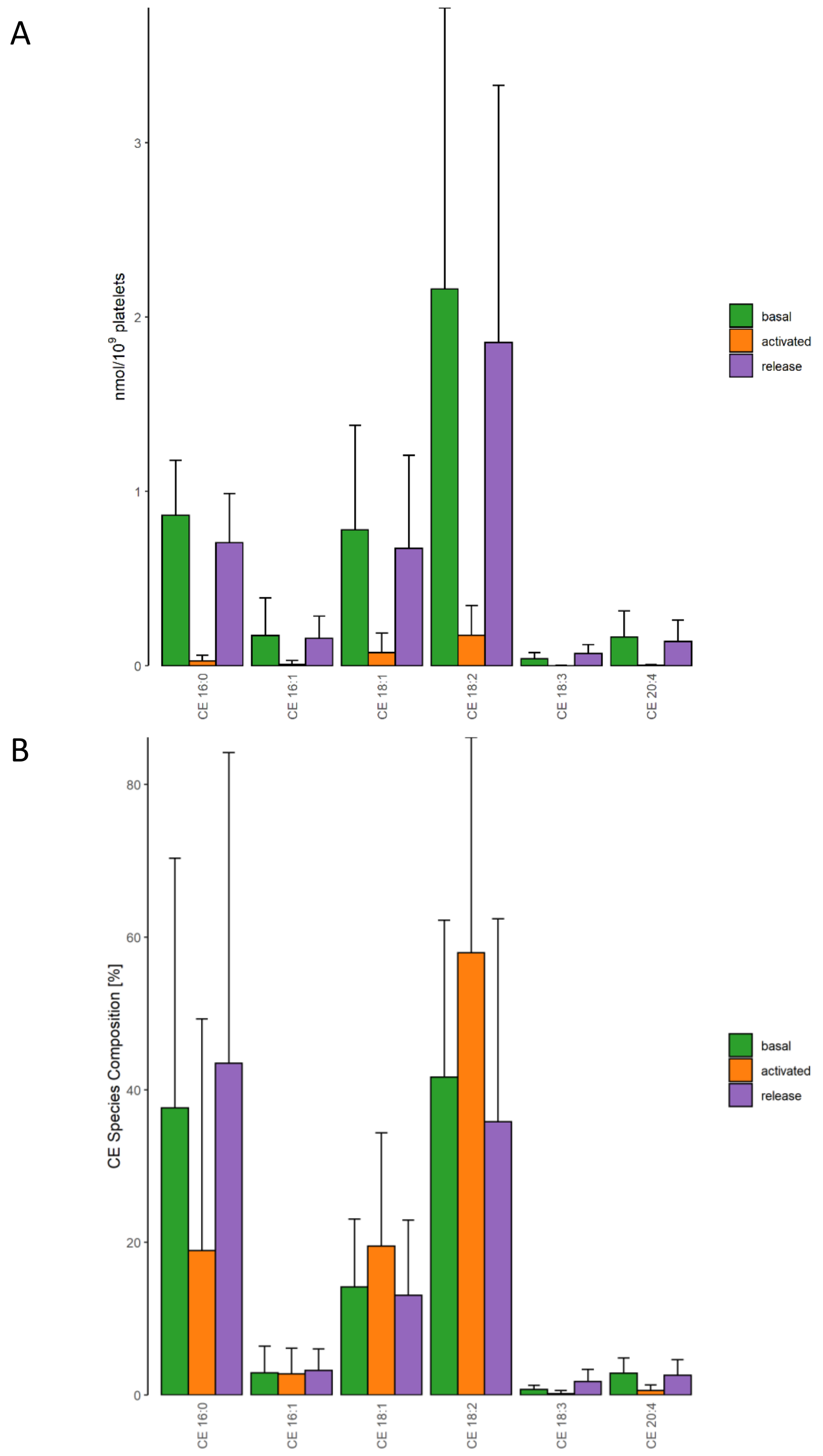

Figure S2. Lipid species concentrations and profiles of CE

Displayed are A) concentrations and B) profiles related to the total CE concentration of unstimulated (basal), activated platelets and lipid release upon thrombin stimulation. Mean and SD of 12 healthy human donors.

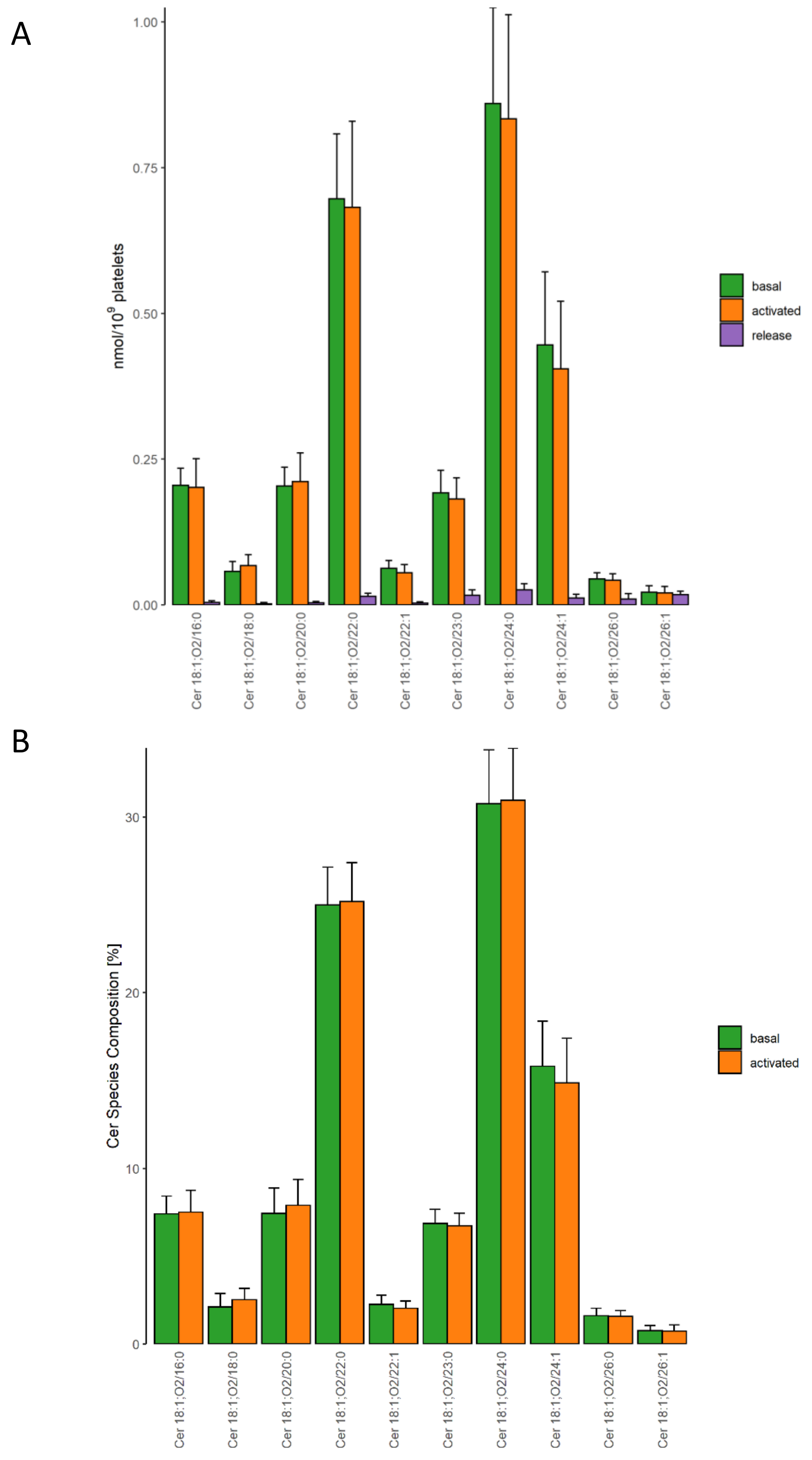

Figure S3. Lipid species concentrations and profiles of Cer

Displayed are A) concentrations and B) profiles related to the total Cer concentration of unstimulated (basal), activated platelets and lipid release upon thrombin stimulation. Mean and SD of 12 healthy human donors.

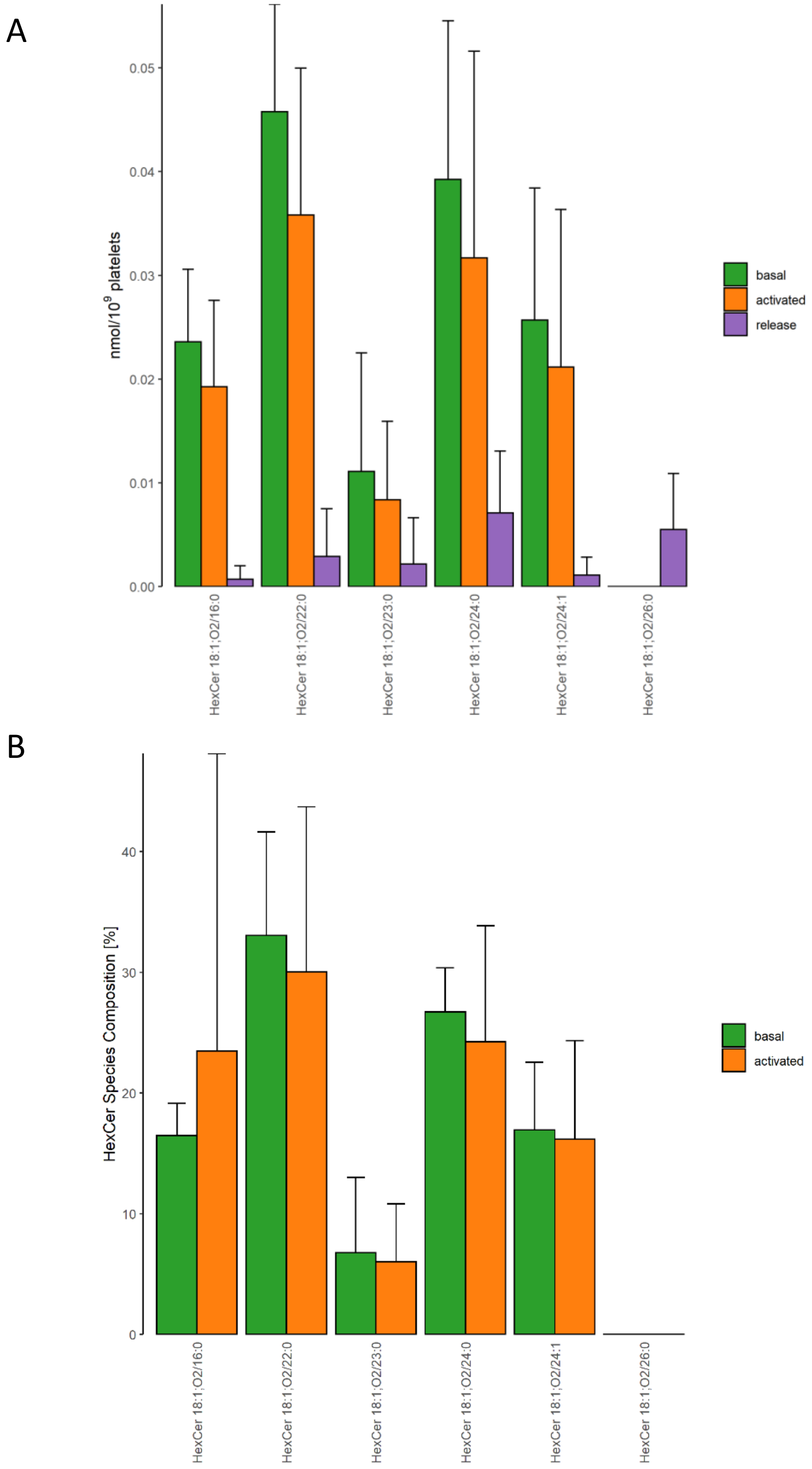

Figure S4. Lipid species concentrations and profiles of HexCer

Displayed are A) concentrations and B) profiles related to the total HexCer concentration of unstimulated (basal), activated platelets and lipid release upon thrombin stimulation. Mean and SD of 12 healthy human donors.

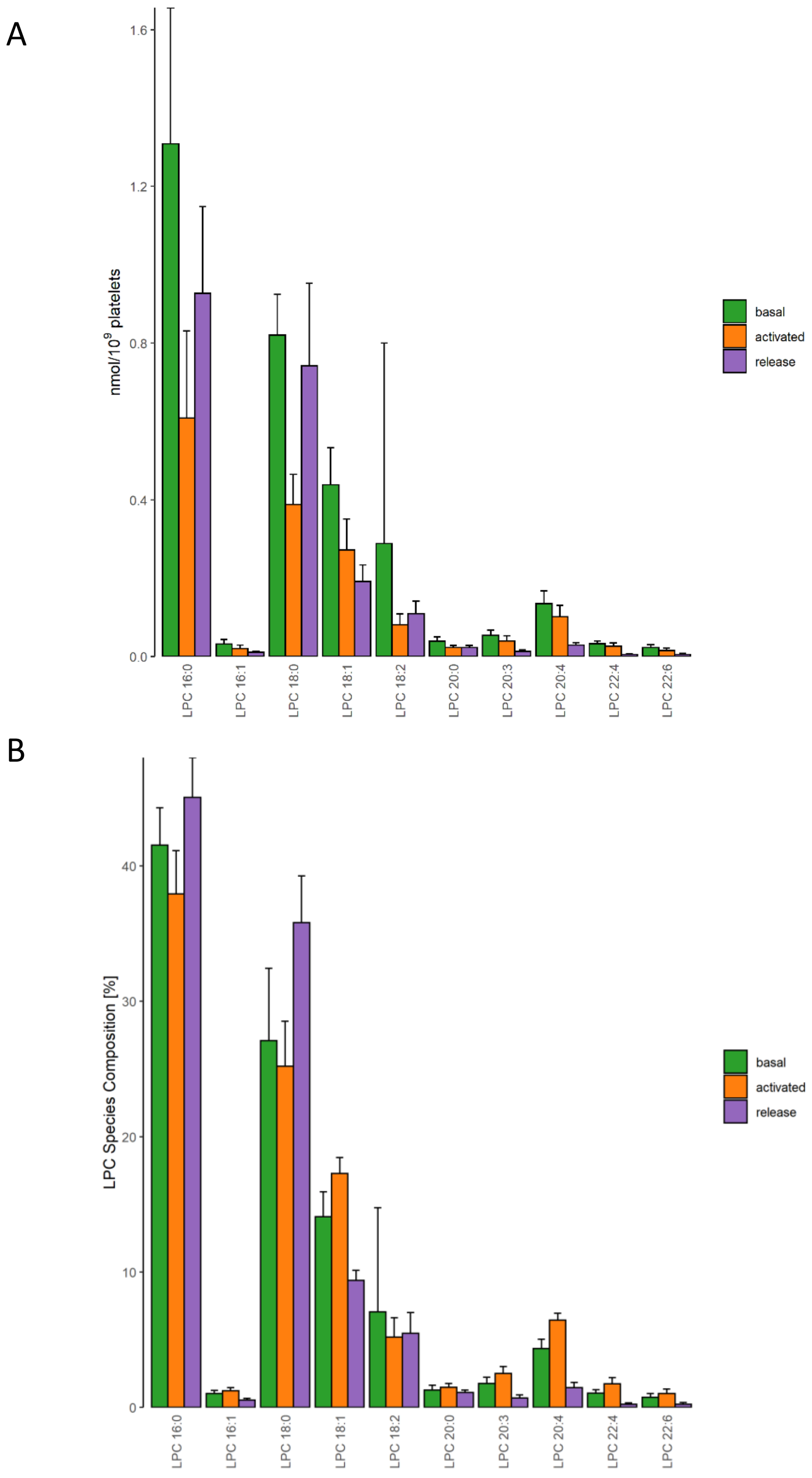

Figure S5. Lipid species concentrations and profiles of LPC

Displayed are A) concentrations and B) profiles related to the total LPC concentration of unstimulated (basal), activated platelets and lipid release upon thrombin stimulation. Mean and SD of 12 healthy human donors.

A

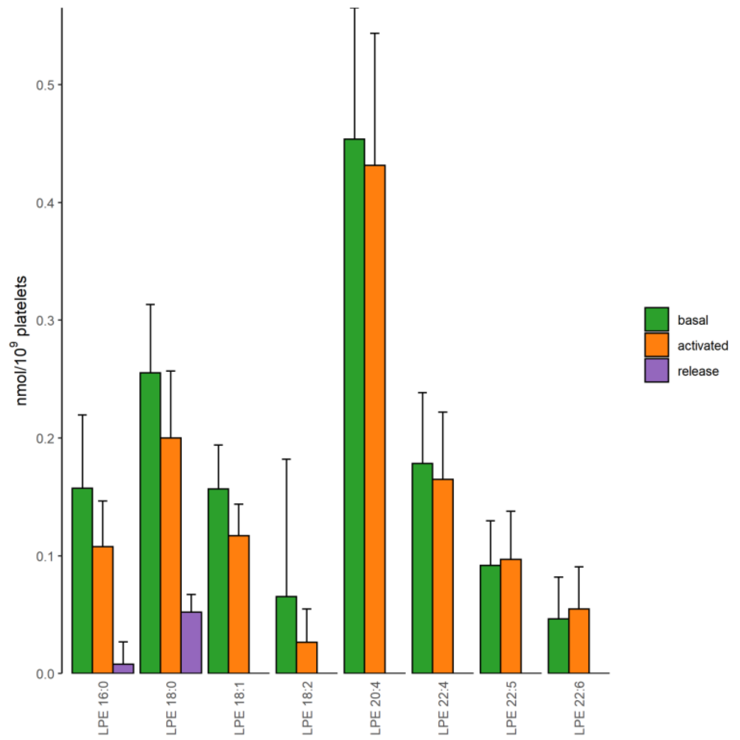

B

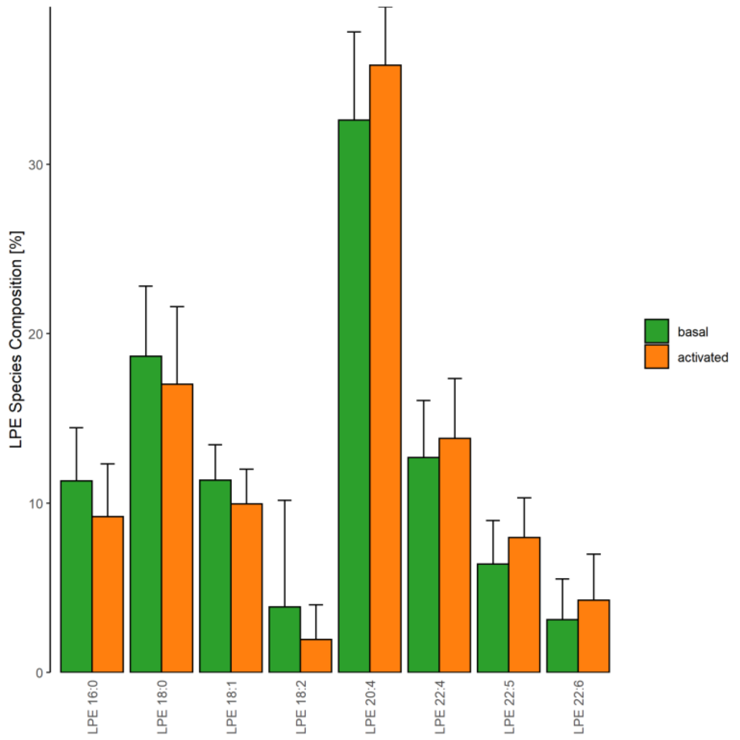

Figure S6. Lipid species concentrations and profiles of LPE

Displayed are A) concentrations and B) profiles related to the total LPE concentration of unstimulated (basal), activated platelets and lipid release upon thrombin stimulation. Mean and SD of 12 healthy human donors.

A

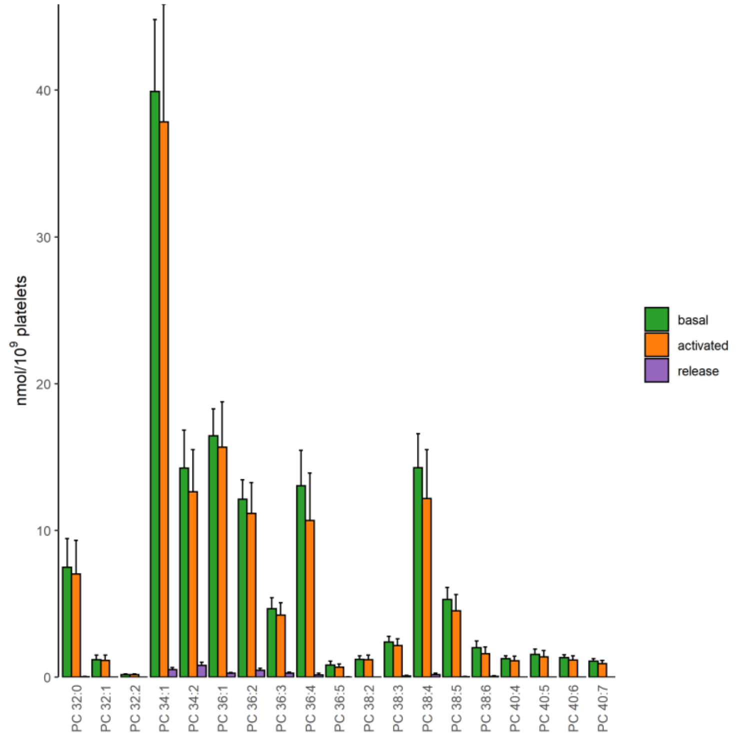

B

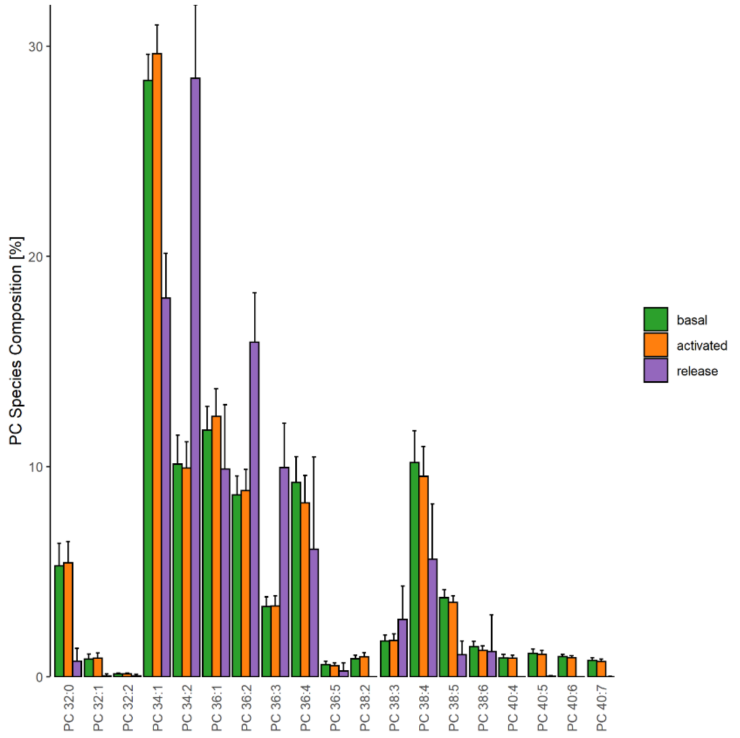

Figure S7. Lipid species concentrations and profiles of PC

Displayed are A) concentrations and B) profile related to the total PC concentration of unstimulated (basal), activated platelets and lipid release upon thrombin stimulation. Mean and SD of 12 healthy human donors.

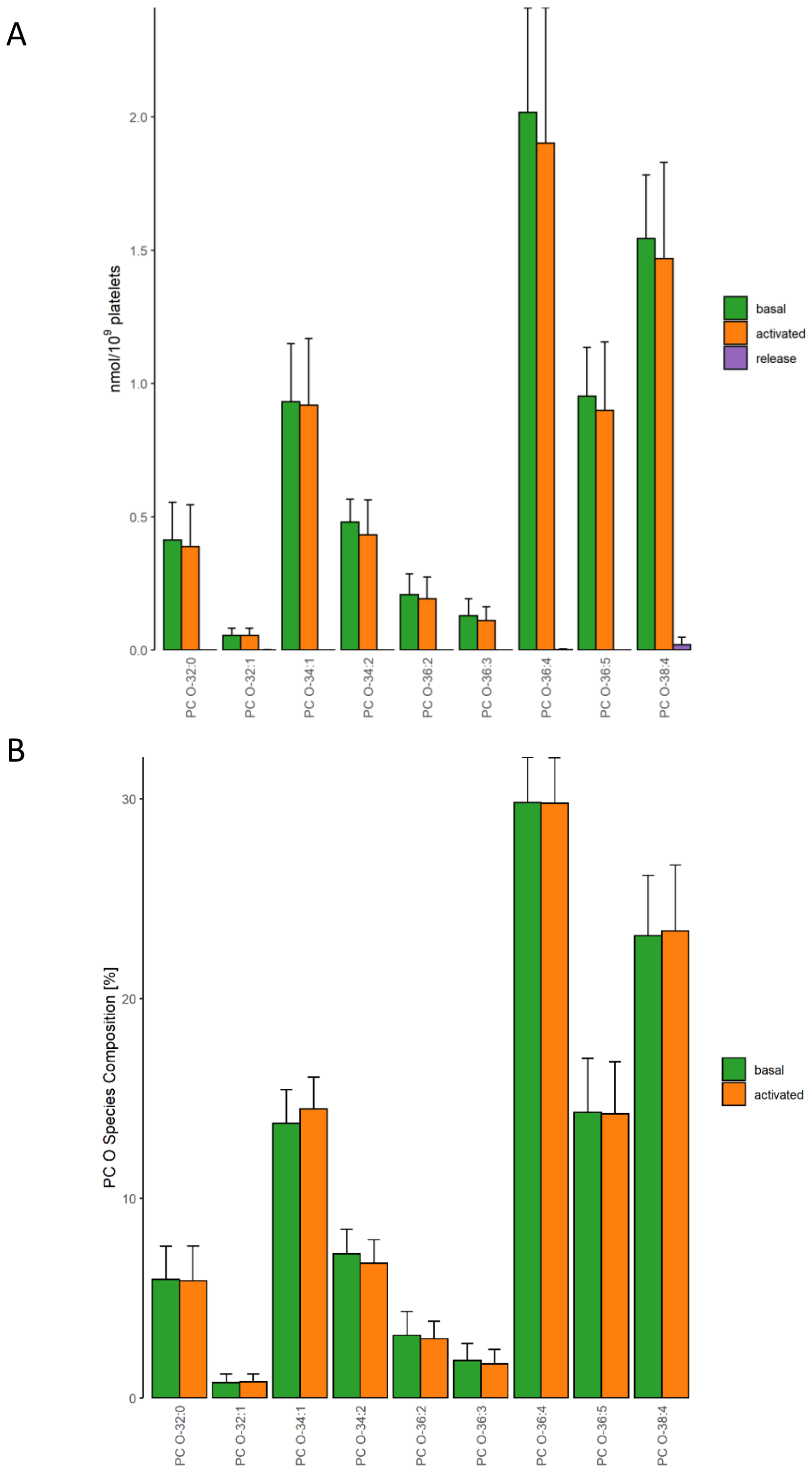

Figure S8. Lipid species concentrations and profiles of PC O

Displayed are A) concentrations and B) profiles related to the total PC O concentration of unstimulated (basal), activated platelets and lipid release upon thrombin stimulation. Mean and SD of 12 healthy human donors.

A

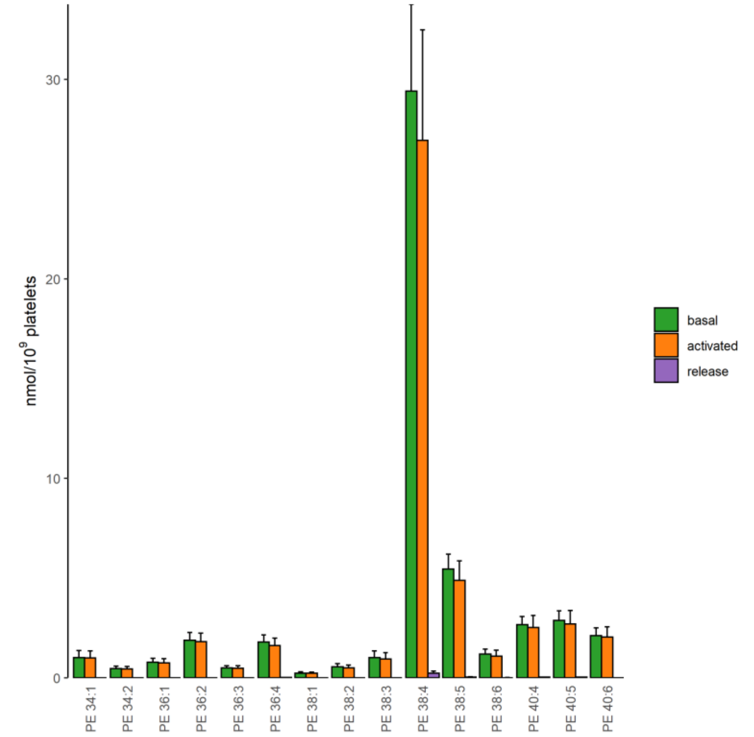

B

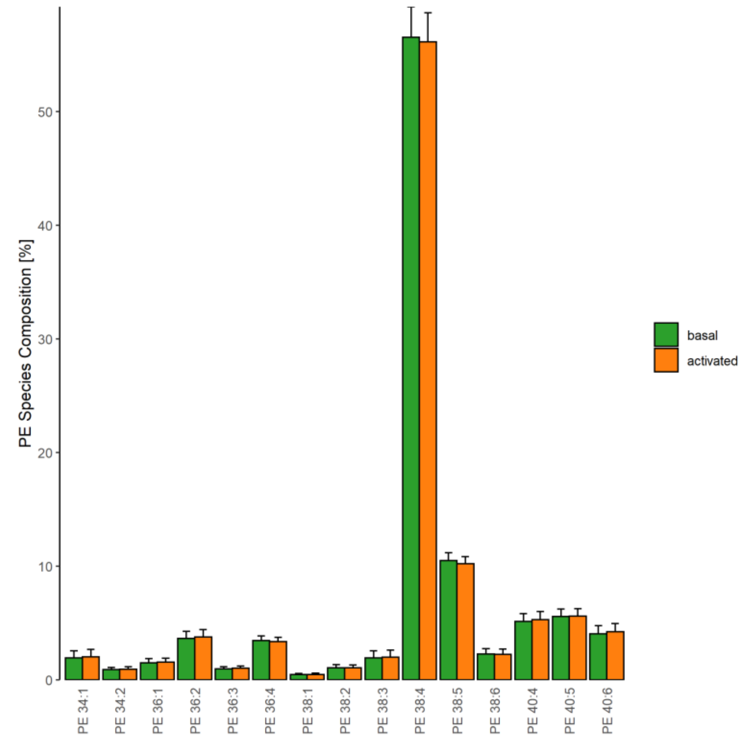

Figure S9. Lipid species concentrations and profiles of PE

Displayed are A) concentrations and B) profiles related to the total PE concentration of unstimulated (basal), activated platelets and lipid release upon thrombin stimulation. Mean and SD of 12 healthy human donors.

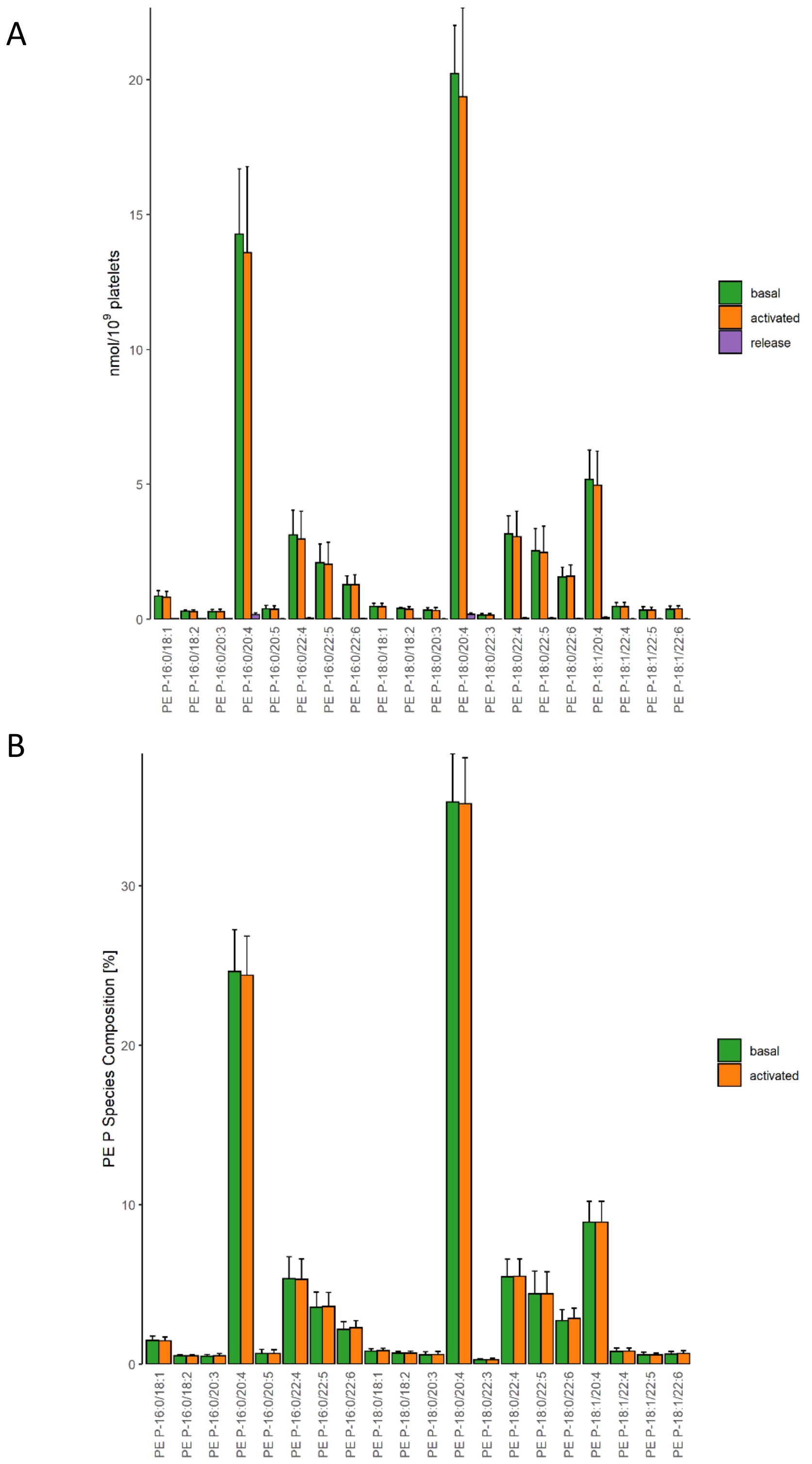

Figure S10. Lipid species concentrations and profiles of PE P

Displayed are A) concentrations and B) profiles related to the total PE P concentration of unstimulated (basal), activated platelets and lipid release upon thrombin stimulation. Mean and SD of 12 healthy human donors.

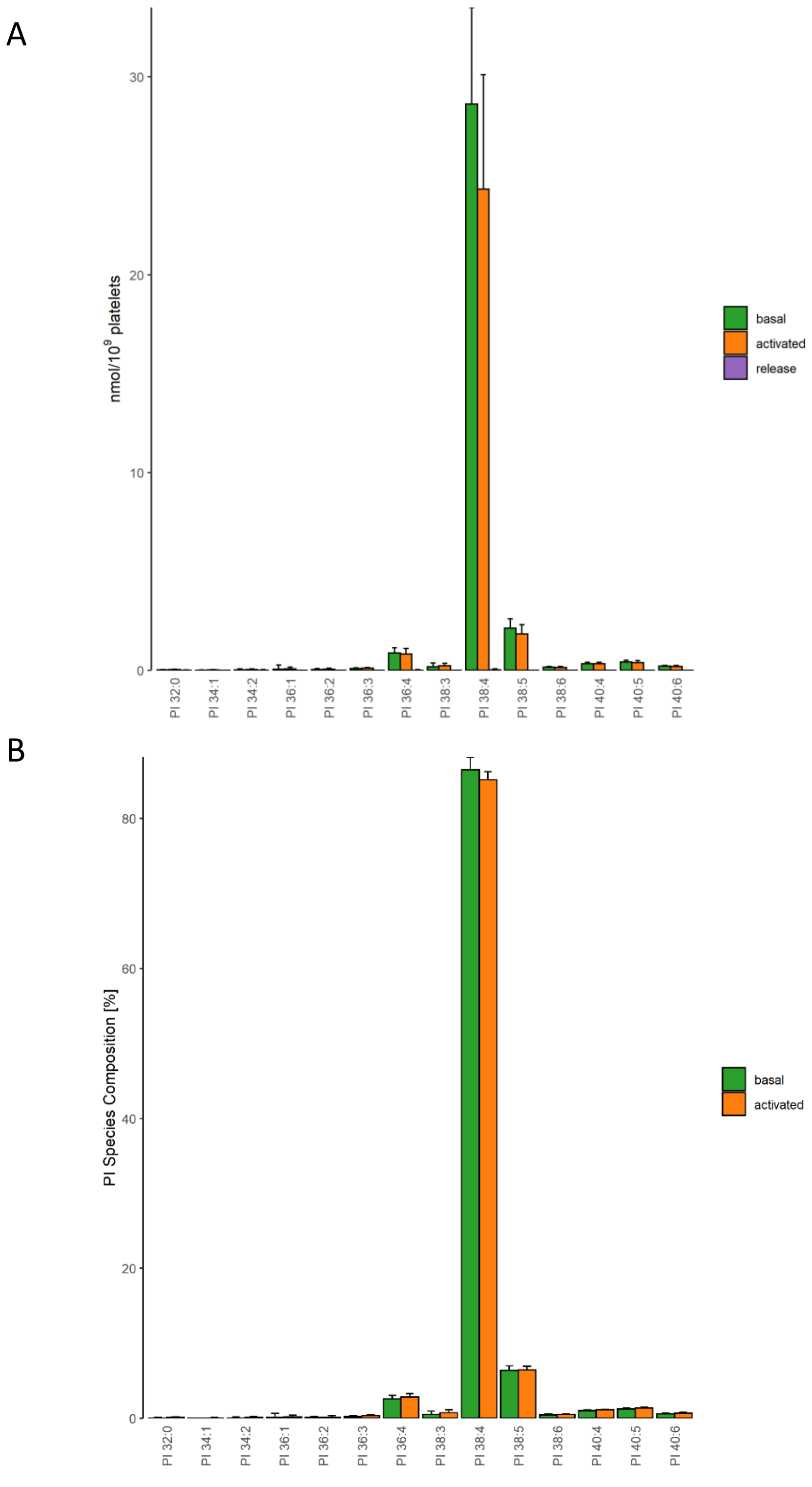

Figure S11. Lipid species concentrations and profiles of PI

Displayed are A) concentrations and B) profiles related to the total PI concentration of unstimulated (basal), activated platelets and lipid release upon thrombin stimulation. Mean and SD of 12 healthy human donors.

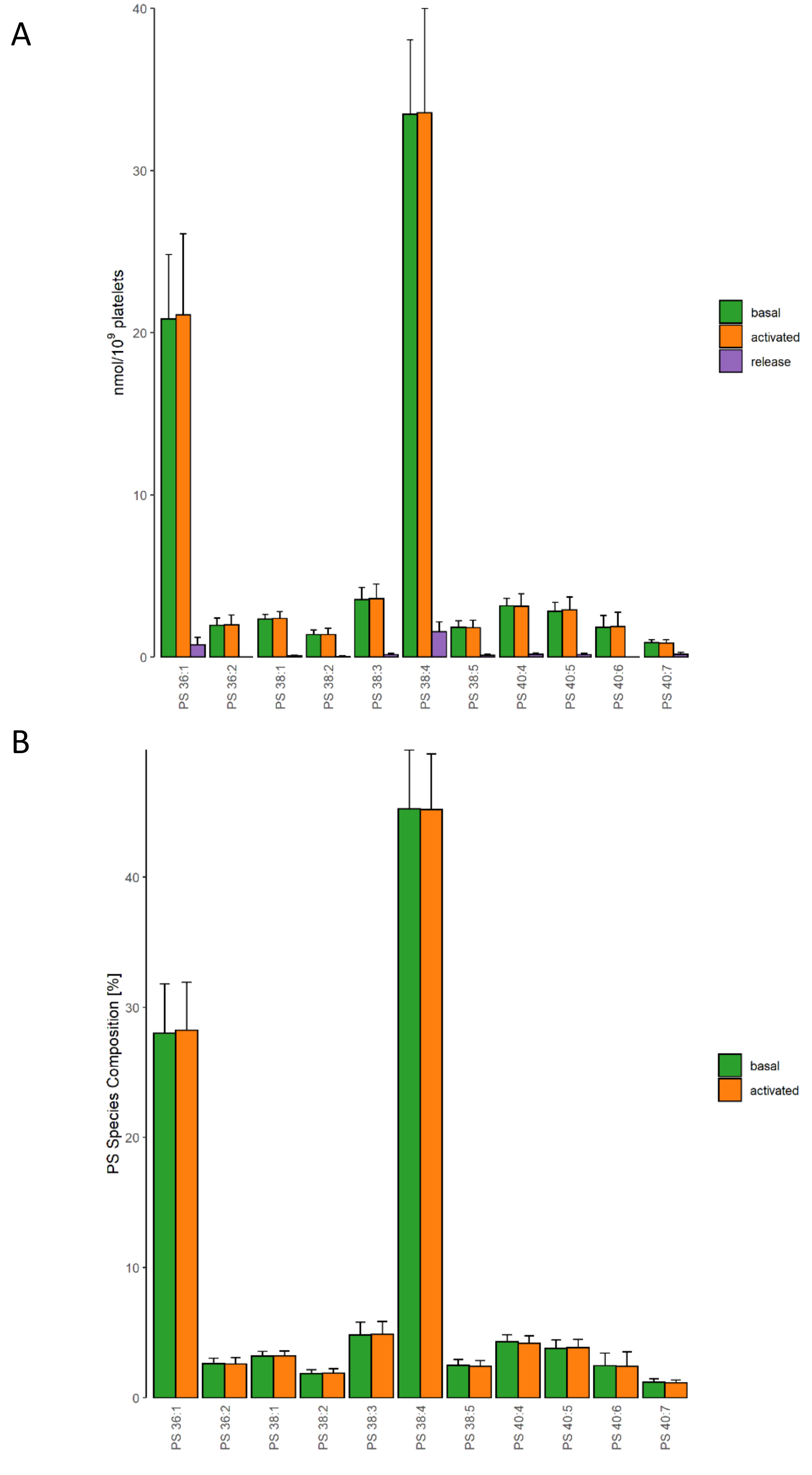

Figure S12. Lipid species concentrations and profiles of PS

Displayed are A) concentrations and B) profiles related to the total PS concentration of unstimulated (basal), activated platelets and lipid release upon thrombin stimulation. Mean and SD of 12 healthy human donors.

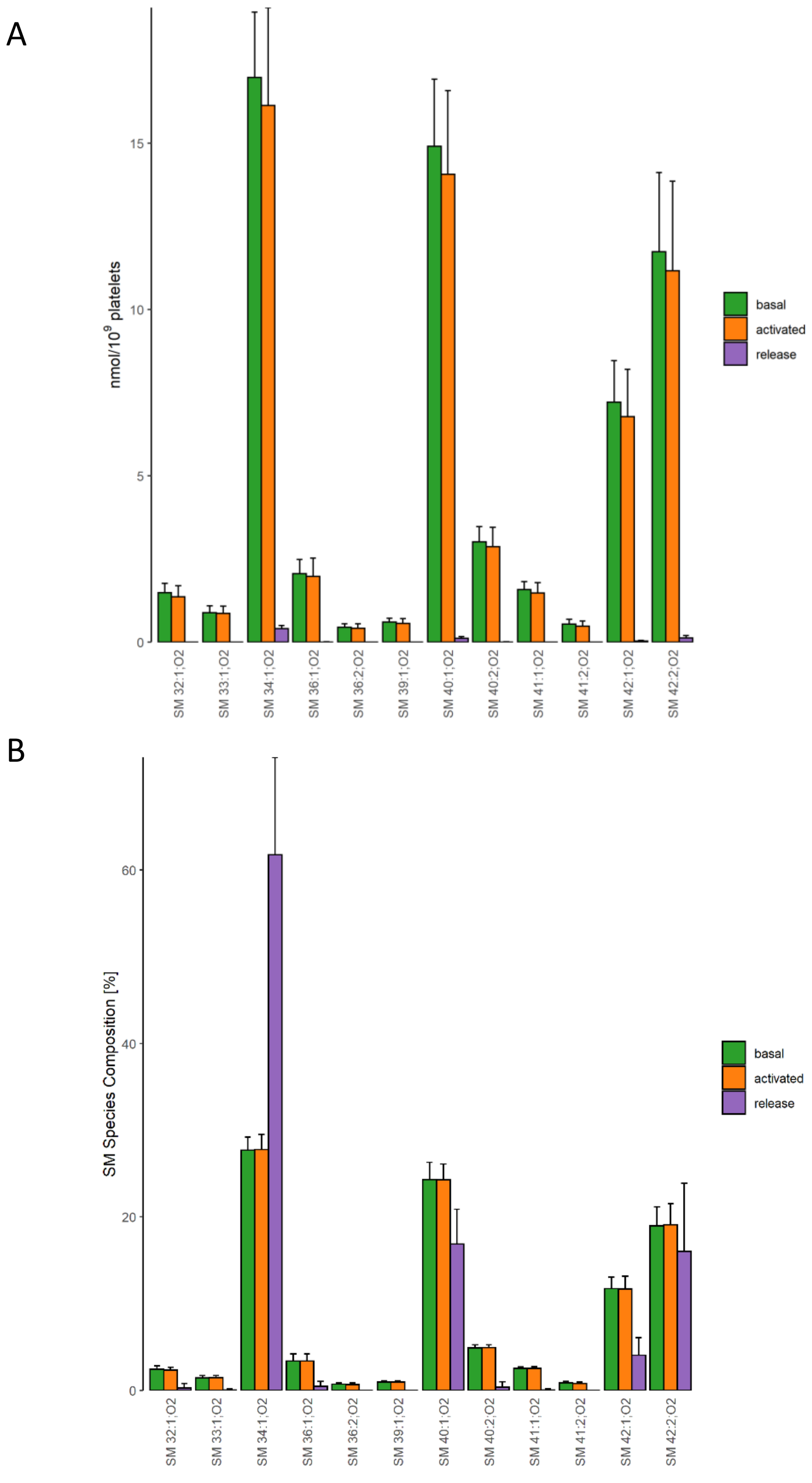

Figure S13. Lipid species concentrations and profiles of SM

Displayed are A) concentrations and B) profiles related to the total SM concentration of unstimulated (basal), activated platelets and lipid release upon thrombin stimulation. Mean and SD of 12 healthy human donors.

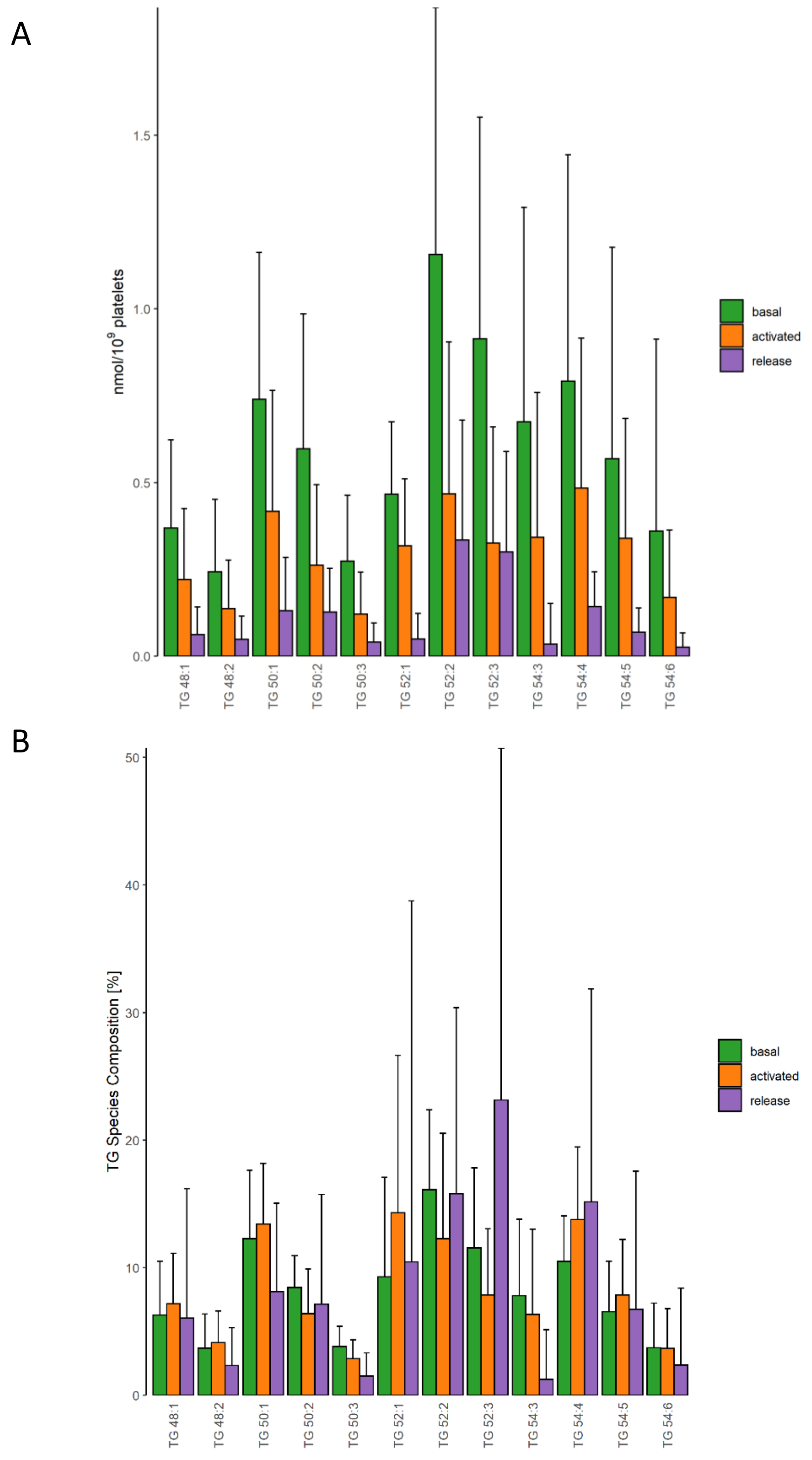

Figure S14. Lipid species concentrations and profiles of TG

Displayed are A) concentrations and B) profiles related to the total TG concentration of unstimulated (basal), activated platelets and lipid release upon thrombin stimulation. Mean and SD of 12 healthy human donors.

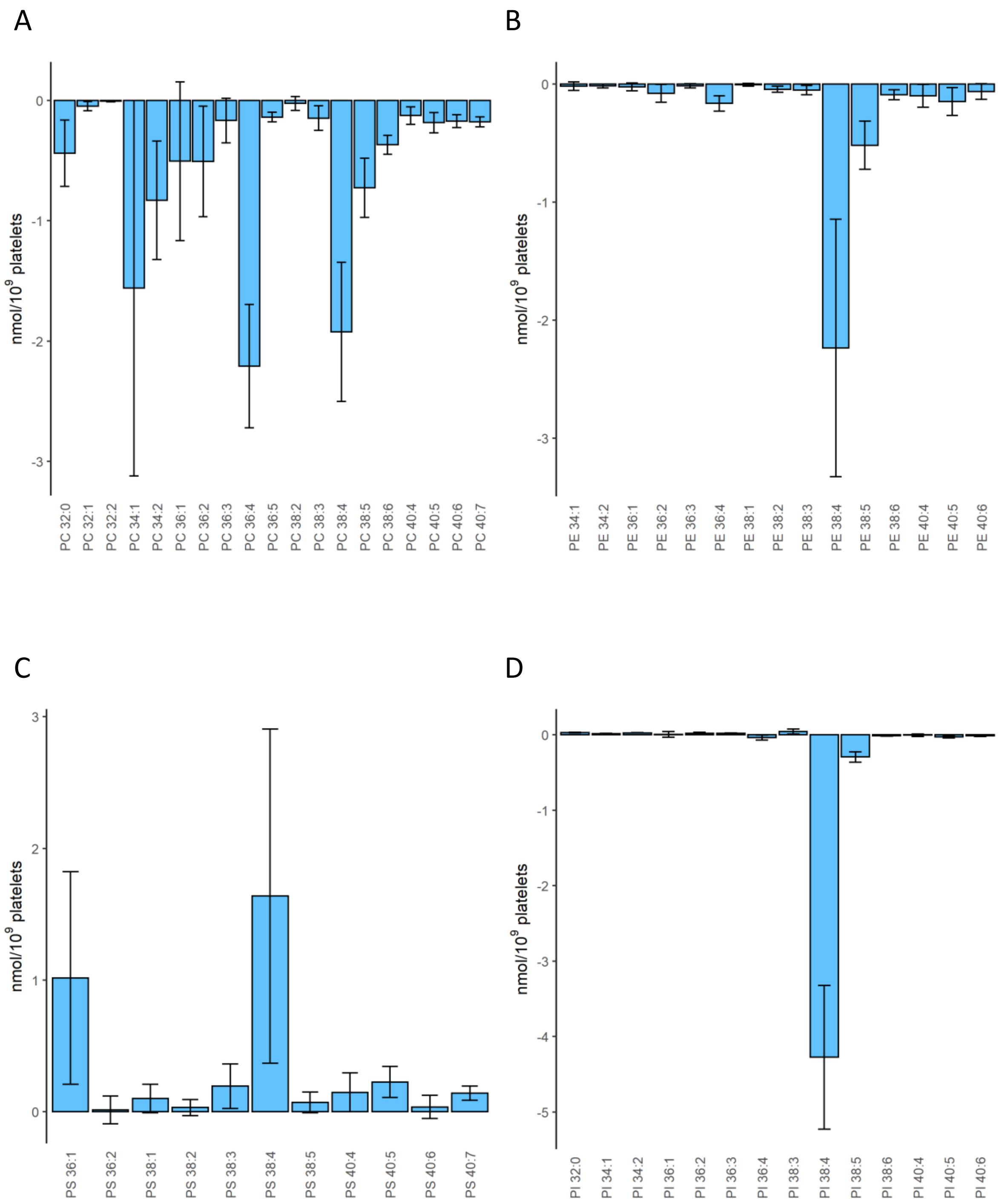

Figure S15. Glycerophospholipid species concentration balance after thrombin activation

Concentration balances were calculated as difference of the sum(lipid release and activated platelets) and basal platelets for the following glycerophospholipid species: A) PC, B) PE, C) PI and D) PS. Displayed are mean and SEM of 12 healthy human donors.
